# Bone-Implant Contact around Crestal and Subcrestal Dental Implants Submitted to Immediate and Conventional Loading

**DOI:** 10.1155/2014/606947

**Published:** 2014-10-14

**Authors:** Ana Emília Farias Pontes, Fernando Salimon Ribeiro, Giovanna Iezzi, Juliana Rico Pires, Elizangela Partata Zuza, Adriano Piattelli, Elcio Marcantonio Junior

**Affiliations:** ^1^Master of Science Program, UNIFEB Educational Foundation of Barretos, Rua Prof. Roberto Frade Monte 389, Aeroporto, Barretos, SP, Brazil; ^2^Department of Oral Health Care Sciences, Dental School, University of Chieti-Pescara (UNICH), Chieti, AB, Italy; ^3^Department of Periodontology, Araraquara Dental School, São Paulo State University (UNESP), Araraquara, SP, Brazil

## Abstract

The present study aims to evaluate the influence of apicocoronal position and immediate and conventional loading in the percentage of bone-implant contact (BIC). Thus, 36 implants were inserted in the edentulous mandible from six dogs. Three implants were installed in each hemimandible, in different positions in relation to the ridge: Bone Level (at crestal bone level), Minus 1 (one millimeter apical to crestal bone), and Minus 2 (two millimeters apical to crestal bone). In addition, each hemimandible was submitted to a loading protocol: immediate (prosthesis installed 24 hours after implantation) or conventional (prosthesis installed 120 days after implantation). Ninety days after, animals were killed, and implant and adjacent tissues were prepared for histometric analysis. BIC values from immediate loaded implants were 58.7%, 57.7%, and 51.1%, respectively, while conventional loaded implants were 61.8%, 53.8%, and 68.4%. Differences statistically significant were not observed among groups (*P* = 0.10, ANOVA test). These findings suggest that different apicocoronal positioning and loading protocols evaluated did not interfere in the percentage of bone-implant contact, suggesting that these procedures did not jeopardize osseointegration.

## 1. Introduction

Over the years, relevant studies have been conducted to investigate the influence of tridimensional implant positioning on esthetic outcomes. Tarnow et al. [1] observed in humans that a remodeling occurs around implant platform, and bone loss occurs in the vertical and horizontal directions, resulting in a saucer or cup shape hard tissue defect. After that, Hermann et al. [2], Todescan et al. [3], and Piattelli et al. [4], based on preclinical studies, concluded that implants inserted apically to crestal bone may present significantly more bone absorption than those inserted more coronally.

Based on these findings, it would seem logical to assume that the optimal treatment plan should include positioning of the top of the implant coronal to bone crest and thus prevent further absorption. However, clinically it could represent a risk for esthetics, as long as the metal prosthetic component or implant platform could become apparent, not to mention that it can lead a poor emergence profile [5]. Garber et al. [6] reinforce that implant placement deeper than usual could be beneficial for aesthetic improvement.

It is interesting to consider that apical positioning could benefit not only esthetics but also bone-implant contact (BIC). Negri et al. [7], Boquete-Castro et al. [8], and Calvo-Guirado et al. [9, 10] inserted implants at crestal level or 2 mm subcrestal, in dogs, under immediate implantation conditions. Then, comparisons were performed with regards to the use of different implant-prosthesis connections, and to different healing periods. Hence, those authors found that around subcrestal group, not only bone remodeling was greater, but also boneimplant contact tended to be higher.

Considering that mechanical loading is another factor that affects bone maintenance [11], Pontes et al. [12, 13] investigated whether biologic width was influenced by apicocoronal position of implants (crestal, 1 and 2 mm apical to the crest) submitting them to immediate or conventional loading. The results suggested that apical positioning of the top of the implant may not jeopardize the position of soft peri-implant tissues and that immediate loading can be beneficial to minimize lateral bone loss. However, the evaluation of BIC was not investigated.

The present study aims to evaluate the influence of apicocoronal position and immediate and conventional restoration in the percentage of BIC.

## 2. Material and Methods

The present study was approved by the Ethical Committee in Animal Research from the State University of São Paulo (protocol number 242003). Six mongrel dogs, featuring good health, weighing 23.0 ± 6.30 kg were included in the present study. Previous to the first surgical intervention, the dogs were submitted to coronal scaling and were molded with condensation silicon.

Thirty-six dental implants (Conect, Conexão Sistema de Prótese Ltda, São Paulo, Brazil) were used in this study (4.3 × 10 mm, sandblasted with titanium oxide, root-form, internal hexagon). In each dog, six dental implants were inserted, three per hemimandible, each one representing an experimental group. The experimental groups were designed according to the distance between the implant abutment junction and the crestal bone: Bone Level group (inserted at crestal bone level), Minus 1 group (one millimeter below crestal bone), and Minus 2 group (two millimeters below crestal bone). Each hemimandible was submitted to a different loading protocol: conventional loading (prostheses installation occurred 120 days after implant placement) or immediate loading (prostheses installation occurred 24 hours after implant placement). Thus, six sets of arrangement were designed, so that an implant representing each group was inserted one time in any site.

In order to carry out surgical procedures, 1% acepromazine (0.02 mg/kg, 0.1 mL/kg, intramuscular) was administered, followed by thiopental (10 mg/kg, 0.5 mL/kg, intravenous). The oral cavity was disinfected with gauzes soaked in 0.12% chlorhexidine solution, and local anesthesia was performed with 2% mepivacaine HCl with norepinephrine 1 : 100.000. An intrasulcular incision was performed, and after the mucoperiosteal flap was reflected, bicuspids were sectioned with high-speed bur under saline irrigation. All mandibular premolars were extracted with forceps, and flaps were closed with 4.0 nylon suture. After the surgical procedures, antibiotic association (penicillin and streptomycin, 24.000 UI/kg, 0.1 mL/kg, intramuscularly) and analgesic ketoprofen (2 mg/kg, 0.4 mL/kg, intramuscularly) were administered. In the following 2 days, the dogs received additional doses of analgesic. During the first week after surgery, the animals were fed a soft diet. Ten days after surgical procedures, sutures were removed. During the experimental period, animals were submitted to a rigorous plaque control with tooth brushing using 0.12% chlorhexidine gel, 3 times a week. These preoperative and postoperative cares were repeated in the following surgical procedures.

After a 90-day period of healing, a crestal incision was performed on the hemimandible designed to be submitted to conventional loading, maintaining similar quantities of keratinized tissue on each side of the incision, and a mucoperiosteal flap was reflected. Dental implants representing each group were inserted, using the mesial crestal bone as reference point. Horizontal distances were determined as follows: 6 mm between the surfaces of adjacent implants and 4 mm between the mesial surface of the first molar and the implant. In sequence, flaps were sutured.

Ninety days afterwards, on the same side, a crestal incision was performed, the cover screws were removed, and healing caps were screwed. The heights of healing caps were selected according to commercial availability, 3 mm, 4 mm, and 5.5 mm, and were used, respectively, in Bone Level, Minus 1 and Minus 2 sites. Then, flaps were closed.

Thirty days after, on the conventional loading side, the healing caps were removed, abutments were placed, and impression was taken using custom-made trays with condensation silicone. On the other side, a crestal incision was performed, the dental implants were inserted, abutments were placed, impression was taken, and flaps were closed. The abutments heights corresponded to those from healing caps.

Twenty-four hours later, metallic fixed partial prostheses were passively screwed. Special attention was taken to avoid occlusal contact. The animals were followed up for 90 days after prostheses installation.

After the animals were killed, mandible and maxilla were dissected, and the specimens were prepared according to a method previously described by Piattelli et al. [14]. The fixation process was accomplished by using 10% neutral formalin for 48 hours. The specimens were dehydrated by using increasing alcohol concentrations, from 60 to 100%. Then, plastic infiltration was processed, with combinations of alcohol and resin (Technovit 7200 VLC. Kulzer, Wehrheim, Germany).

The specimens were polymerized, sectioned at about 150 *μ*m using a specific system (Precise 1 Automated System, Assing, Rome, Italy), and ground down to about 100 *μ*m. Slides were stained with toluidine blue and acid fuchsine and were analyzed using a microscope connected to a video camera interfaced to a computer, where specific processing software was used for measurements (ImageJ 1.34, National Institutes of Health, Bethesda, MA, USA). Images were measured with regard to the percentage of bone-implant contact all around implant body.

All the 36 implants were available for data collection. Values were expressed in means, and the unit of analysis was the dog. Experimental data was submitted to a normality test (Shapiro-Wilk), followed by analysis of variance (ANOVA) test. The null hypothesis was based on the absence of differences among the modalities of treatment (*α* = 5%).

## 3. Results

**Figure 1:**
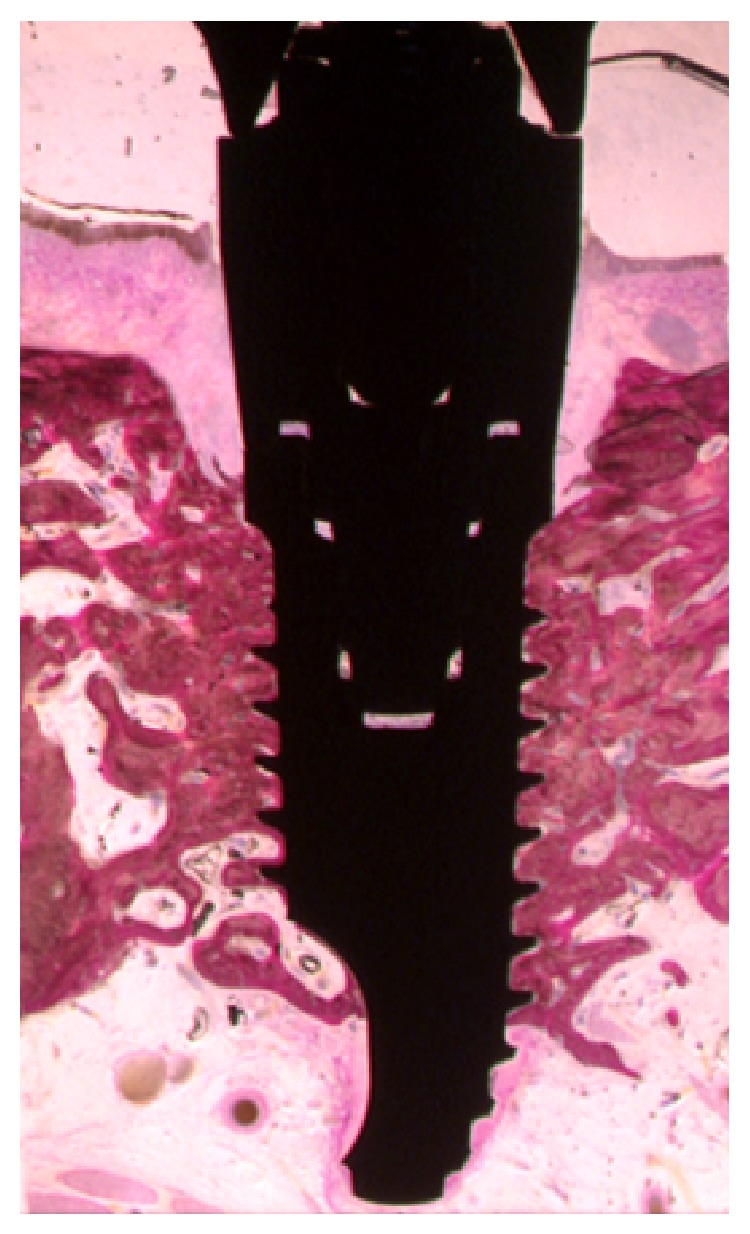
Histometric analysis in the percentage of bone-implant contact was evaluated. In this case, the calculated percentage was 57.6%.

Healing was uneventful in all animals, no loss of either implants or prostheses was observed during the experimental period, and a direct contact was observed between living bone and all implants without interposed soft tissues at the light microscope level (Figure 1).

**Figure 2:**
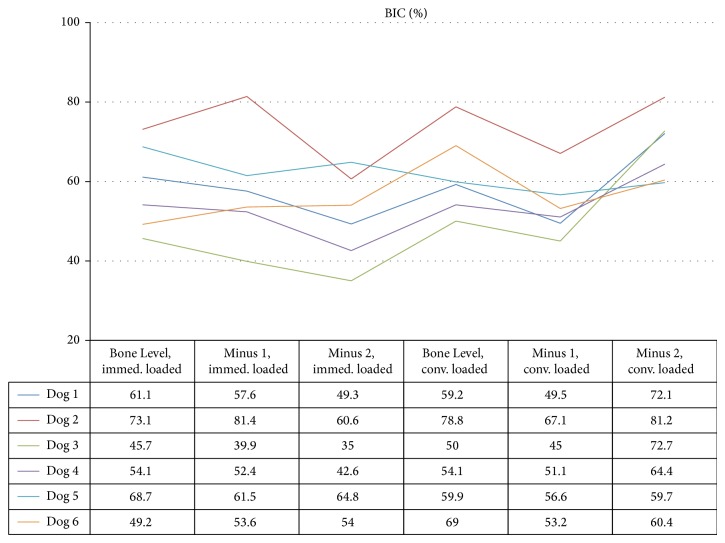
BIC values (%) 90 days after loading for each dog. Immed. loaded = immediately loaded; conv. loaded = conventionally loaded. Statistically significant differences were not observed among groups (*P* = 0.10, ANOVA test).

Mean (±standard deviation) BIC values from immediate loaded implants were 58.7 ± 10.9%, 57.7 ± 13.7%, and 51.1 ± 11.1%, respectively, for Bone Level, Minus 1, and Minus 2 groups, while conventional loaded implants were 61.8 ± 10.5%, 53.8 ± 7.6%, and 68.4 ± 8.4% (Figure 2). Differences statistically significant were not observed among groups (*P* = 0.10, ANOVA test).

In all groups, the presence of compact bone was observed and evenly distributed around the entire implant surface, from the coronal portion to the apex of the implant; and there was no interposition of fibrous tissue interface bone-implant.

In Bone Level group submitted to immediate loading, at the apex of the implant, where bone usually tends to be more trabecular, compact bone was present. Some threads were surrounded by marrow spaces where there was an intense osteoblastic activity.

In Minus 1 group submitted to immediate loading, the presence of compact bone was observed especially at the coronal and medial portion of the implants. New bone formation with osteoblastic activity was detected in the coronal portion of the implant.

In Minus 2 group submitted to immediate loading, bone remodeling occurred at the coronal portion of the implants; in a general manner, bone had a solid and mature aspect.

In Bone Level group submitted to conventional loading, the threads of the implants were surrounded by newly formed bone tissue. Implants were surrounded by trabecular bone with wide marrow spaces, and near the surface of the implant, bone was more compact and marrow spaces were smaller.

In Minus 1 group submitted to conventional loading, the presence of bone tissue was observed, especially at the coronal third and medium. A compact bone was present, with small marrow spaces and secondary osteons. Adjacent to the threads, there were numerous areas of bone remodeling. Trabecular bone with wide marrow spaces was observed especially at the middle and apical third of the plant.

In Minus 2 group submitted to conventional loading, the presence of new bone formation was detected in the coronal portion of the implant. The implants were surrounded by trabecular bone with wide marrow spaces, especially in the apical portion.

## 4. Discussion

This study evaluated bone-implant contact of implants inserted under different clinical conditions. The implication is that, at least under the conditions studied, submerging two-piece implants and submitting to immediate or conventional loading do not necessarily jeopardize osseointegration.

The methodology of this study was designed to clarify whether the osseointegration of the implants would be influenced by the absorption of crestal bone that occurred adjacent to the platform of the implant or by the low bone density observed mainly in the most apical portion of those implants inserted apically to the crestal bone.

Different loading protocols were evaluated, because mechanical loading influences bone remodeling [11], and this variable was not considered in the studies from Hermann et al. [2], Todescan et al. [3], Negri et al. [7], Boquete-Castro et al. [8], and Calvo-Guirado et al. [9, 10], in which prostheses were not installed.

BIC values ranged from 51.1% to 69.4%, and these values seem to be enough to warrant stability, since values were higher than 50% [15]. In the study from Todescan et al. [3], mean BIC values were the following: 46.8% for implants inserted 1 mm coronal to the crest, 53.7% at crestal position, and 49.0% 1 mm apical to the crest. Such values, lower than those from present study, may be justified by lower mechanical stimulus.

Similarly, Boquete-Castro et al. [8] developed a study in which BIC of 53.85% was reported for 2 mm subcrestal group and 39.50 ± 9.25% for crestal group. Then, Calvo-Guirado et al. [9] reported that BIC values tended to increase in 2 mm subcrestal group (47.33% at 8 weeks and 53.85% at 12 weeks) and to decrease in crestal group (44.52% at 8 weeks and 39.50% at 12 weeks).

On its turn, Calvo-Guirado et al. [10] evaluated different implant geometry (external hexagon, internal hexagon, and internal conical connection). Implants inserted 2 mm subcrestally resulted in 47.33 ± 5.23%, 48.38 ± 11.63%, and 54.88 ± 11.73.%, while those inserted at crestal level resulted in 42.52 ± 8.67%, 35.19 ± 18.12%, and 47.46 ± 11.50%, respectively. Differences between each subgroup in the test and the control groups were statistically significant.

Negri et al. [7] compared bone-implant contact from different implant designs. Tapered and cylindrical implants resulted in 33.85 ± 5.21% and 45.87 ± 2.02% in subcrestal group and 29.50 ± 9.25% and 42.52 ± 8.78% for crestal group, respectively. Thus, there was less bone resorption in the subcrestal group than crestal group. Additionally, cylindrical implants, as those used in present study, led to higher BIC values.

Present study and those from Boquete-Castro et al. [8], Calvo-Guirado et al. [9, 10], and Negri et al. [7] corroborate the arguments from Garber et al. [6], who suggested that two-piece implants may be successfully indicated.

In the present study, developed in posterior mandible, large mongrel dogs were used, because it was necessary to install commercially available implants even 2 mm apical to crestal bone and screw their abutments and prosthesis. A split-mouth design and random selection of groups were performed in order to reduce bias; moreover, histologic evaluation, which is considered as a gold standard, was carried to clarify precisely newly formed tissues.

However, main limitations of this study were that implants were installed neither in esthetic area nor in humans. Implants were not installed in esthetic zone because it was important to insert implants in an area long enough to support six implants, in a flat alveolar bone, which was achieved after surgical extraction of the premolars and regularization of the posterior border of the mandible. Secondly, a preclinical model was preferred to limit the variable involved and to allow histologic evaluation.

Future studies with longer healing periods and human clinical and radiographic trials should be conducted to provide data concerning the stability of the implants to support these findings and evaluate clinical outcome after the insertion of implants in the esthetic zone, where bone tends to assume a scalloped shape.

## 5. Conclusions

Within the sample studied, it could be concluded that different apicocoronal positioning and the restoration protocols evaluated did not interfere in the percentage of BIC, suggesting that these procedures did not jeopardize its osseointegration.
